# A Novel Method for Screening Adenosine Receptor Specific Agonists for Use in Adenosine Drug Development

**DOI:** 10.1038/srep44816

**Published:** 2017-03-20

**Authors:** Karlie R. Jones, Uimook Choi, Ji-Liang Gao, Robert D. Thompson, Larry E. Rodman, Harry L. Malech, Elizabeth M. Kang

**Affiliations:** 1 Laboratory of Host Defenses, National Institute of Allergy and Infectious Diseases, National Institutes of Health, Bethesda MD 20892, USA; 2 Molecular Signaling Section, Laboratory of Molecular Immunology, National Institute of Allergy and Infectious Diseases, National Institutes of Health, Bethesda MD 20892, USA; 3 Lewis and Clark Pharmaceuticals Inc., Charlottesville, VA 22901, USA

## Abstract

Agonists that target the A_1_, A_2A_, A_2B_ and A_3_ adenosine receptors have potential to be potent treatment options for a number of diseases, including autoimmune diseases, cardiovascular disease and cancer. Because each of these adenosine receptors plays a distinct role throughout the body, obtaining highly specific receptor agonists is essential. Of these receptors, the adenosine A_2A_R and A_2B_R share many sequence and structural similarities but highly differ in their responses to inflammatory stimuli. Our laboratory, using a combination of specially developed cell lines and calcium release analysis hardware, has created a new and faster method for determining specificity of synthetic adenosine agonist compounds for the A_2A_ and A_2B_ receptors in human cells. A_2A_ receptor expression was effectively removed from K562 cells, resulting in the development of a distinct null line. Using HIV-lentivector and plasmid DNA transfection, we also developed A_2A_ and A_2B_ receptor over-expressing lines. As adenosine is known to cause changes in intracellular calcium levels upon addition to cell culture, calcium release can be determined in these cell lines upon compound addition, providing a functional readout of receptor activation and allowing us to isolate the most specific adenosine agonist compounds.

Adenosine receptors play a regulatory role throughout the body, having been shown to be active in virtually every organ system studied. Extracellular adenosine accumulated in response to metabolic stress and/or cell damage has one of four effects on tissue protection and repair: (a) increasing oxygen supply to damaged tissues; (b) protection against ischemic damage due to cell conditioning; (c) countering inflammatory responses; (d) and promoting angiogenesis^1^,^2^,^3^,^4^. Due to adenosine’s essential role in modulating tissue homeostasis, it is also important in a number of diseases, including neurological disorders, cardiovascular disease and autoimmune responses in the body^5^,^6^,^7^.

Adenosine modulates these different effects through binding to one or more of its four G protein coupled receptors (A_1_, A_2A_, A_2B_ and A_3_). Adenosine binding with the A_1_ receptor decreases heart rate and neuronal activity, while all adenosine receptors have roles in many types of cancer^4^,^8^,^9^,^10^,^11^,^12^. In general, anti-inflammatory aspects of adenosine are associated with its binding to the A_2A_ receptor (A_2A_R)^13^,^14^,^15^; alternatively, adenosine binding to the A_2B_ receptor (A_2B_R) has been associated with pro-inflammatory actions of adenosine^16^,^17^,^18^. The adenosine A_2A_R and A_2B_R share many sequence and structural similarities (Fig. 1) but have opposing immunological responses to inflammatory stimuli, indicating that therapies targeting either the A_2A_R or A_2B_R need to be very specific to achieve the desired therapeutic effect^19^,^20^.

Current methods of determining receptor activity and binding of adenosine analogues for therapeutic use against specific receptors are ponderous and time-consuming. These methods often involve the use of radio-ligand binding assays followed by competition and saturation binding experiments. This is typically followed by toxicity assays in cell culture and animal models to assess safety and efficacy. Alternatively, analysis of analogues for receptor affinity by hit identification using computational software is faster and safer, but less reliable for true biological effects of the analogue molecule in the cell. Further, computational analysis must be followed by *in vitro* and *in vivo* toxicity experiments. Thus development of a new method for quick and safe characterization of novel adenosine analogues is needed to streamline adenosine drug development.

Our laboratory has designed a new method of A_2A_R and A_2B_R agonist analogue characterization utilizing engineered cell lines and calcium flux assays, a method that can be expanded to incorporate additional adenosine receptors. Using Clustered Regularly Interspaced Short Palindromic Repeats (CRISPR) technology for targeted genome editing, A_2A_R expression was knocked out in the K562 cell line. Additionally, because of the homology of the A_2A_R and A_2B_R and the potential for adenosine analogues to bind indiscriminately between the two, A_2A_R and A_2B_R over-expressing K562 lines were created utilizing HIV-lentivector. As adenosine is known to cause changes in intracellular calcium levels upon addition to cell culture, calcium flux assays were employed with these cell lines to determine specificity^21^,^22^,^23^,^24^. Using a microplate reader, we were able to read changes in intracellular free calcium levels upon compound addition to both our knockout and over-expressing lines, allowing us to determine off-target binding of A_2A_R and A_2B_R agonists of interest. Those compounds found to be specific to the A_2A_R are then screened for efficacy using human naïve T-cells, evaluating them on their ability to induce differentiation of FoxP3^+^ regulatory T-cells, a cell type associated with immune tolerance and anti-inflammatory responses^14^. Overall, this new method will allow for faster characterization of adenosine receptor binding partners and will accelerate drug discovery.

## Results

### Development of a CRISPR/Cas9 Vector for Knockout of the A_2A_R in K562 Cells

Recently, CRISPR/Cas9 has emerged as an efficient and reliable way to perform targeted genome editing^25^. Delivery of the Cas9 nuclease and appropriate gRNAs into a cell allows the cell’s genome to be cut at a specific location, inactivating existing genes or adding new ones. The human hematopoietic tumor line K562 is a non-adherent easily maintained cell line convenient for our studies because it natively expresses low levels of the A_2A_R and also has almost no expression of the A_2B_R. To create a line that strongly expresses the A_2B_R we developed a CRISPR/Cas9 strategy to engineer a K562 line in which A_2A_R expression was fully knocked out.

**Figure 2:**
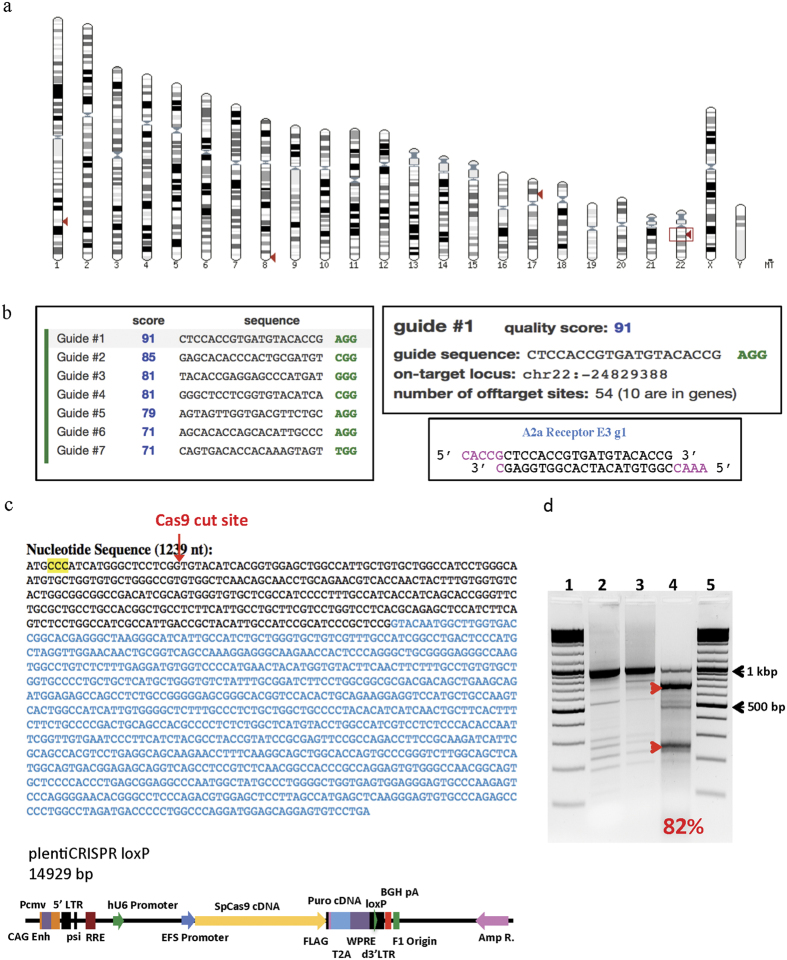
CRISPR vector design for knockout of A_2A_R in K562 Cells. (**a**) Map of human chromosomes, with chromosome 22q11.23 highlighted, showing the location of the A_2A_R gene. Additional arrowheads indicate genes with similar regions to the A_2A_R gene, including the A_2B_R gene on chromosome 17. (**b**) Left, guide-RNAs found for the A_2A_R gene. Right, guide sequence #1 was selected for insertion into the plentiCRISPR loxP vector plasmid DNA. BsmBI cut sites were added to gRNA #1 to facilitate insertion. (**c**) Top, Cas9 cut site in the A_2A_R gene. Bottom, map of the plentiCRISPR construct. (**d**) T7 E1 INDEL assay for CRISPR targeting efficiency. Lanes 1 and 5, universal marker. Lane 2, PCR product. Lanes 3 and 4, PCR product after denaturing and renaturing, without (3) and with (4) T7 EI enzyme. Arrowheads indicate T7 E1 cutting.

The A_2A_R gene is located on chromosome 22q11.23 and is approximately 2.6 kbp long (Fig. 2a). Several gRNA sequences for CRISPR/Cas9 mediated knockout were found for A_2A_R with varying quality scores (Fig. 2b, left). gRNA #1 (5′-CTCCACCGTGATGTACACCG-3′) was selected as it has a quality score of 91, indicating minimal off-target sites (Fig. 2b, upper right). BsmBI cut sites were added to gRNA #1 for insertion into the plentiCRISPR loxP vector plasmid DNA (Fig. 2b, bottom right). Figure 2c shows the plentiCRISPR loxP construct and cut site on the A_2A_R gene. Cas9 cutting is targeted to exon 1 of the A_2A_R gene (black letters) rather than exon 2 (blue letters) to ensure efficient knockout of the gene. Using the T7 E1 enzyme for insertion and deletion (INDEL) assay, an indicator of CRISPR targeting efficiency, we observed 82% of transduced K562 cells with the modified A_2A_R locus, indicating high targeting efficiency of the plentiCRISPR loxP A_2A_R virus (Fig. 2d).

### Creation of a Stable A_2A_ Receptor Knockout Line in K562 Cells

**Figure 3:**
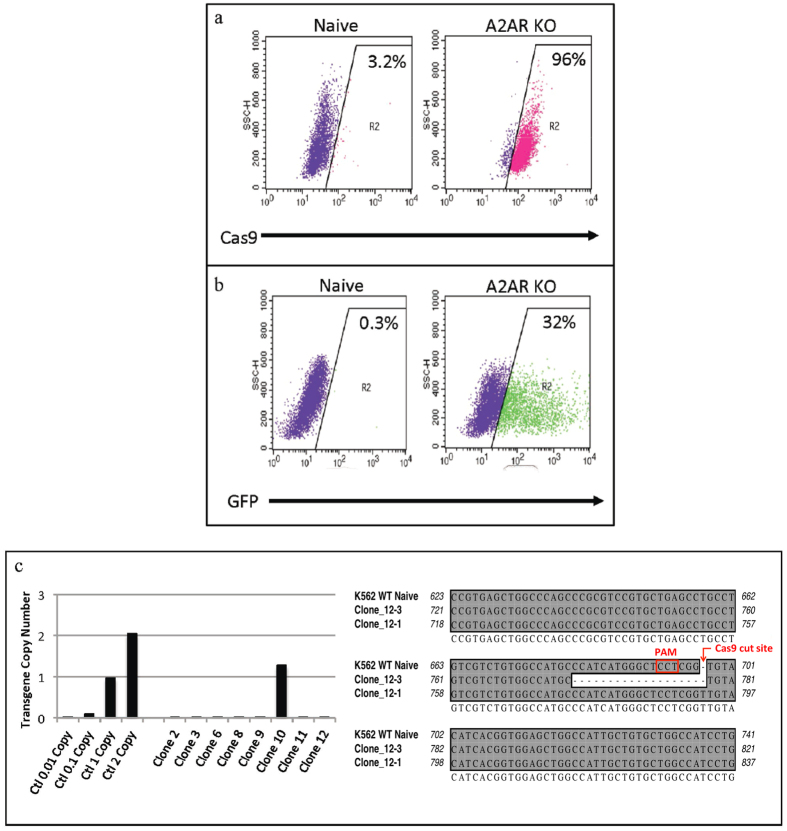
Development of a stable A_2A_R knockout cell line in K562 cells using CRISPR. (**a**) Flow cytometry for Cas9 expression in K562 cells before and after transduction with the plentiCRISPR loxP A2AR. After puromycin selection to remove non-transduced cells, 96% of cells were successfully transduced. (**b**) Flow cytometry for removal of the loxP cassette using the pcDNA3.1 ZEO T2A CRE-GFP plasmid. 32% of cells express GFP, indicating successful removal of the proviral vector cassette. These cells were further selected using zeocin for isolation of cassette free cells. (**c**) Left, RT-PCR of clones selected from loxP cassette-free A_2A_R knockout K562 cells. Right, sequencing of clone 12 reveals a one base (T) insertion on one allele and an 18 base deletion on the other for homologous knockout of the A_2A_R gene.

The plentiCRISPR loxP A_2A_R virus was transduced into K562 cells to produce a stable A_2A_R knockout line. Flow cytometry on transduced cells for efficiency of transduction showed 96% of cells successfully transduced after puromycin selection (Fig. 3a). After selection, the A_2A_R knockout K562 cells were transfected with the pcDNA3.1 ZEO T2A CRE-GFP plasmid to remove the loxP cassette. The proviral vector cassette was successfully removed from 32% of transfected cells as shown by flow cytometry (Fig. 3b). Cells were then selected by zeocin for isolation of cells without cassettes. Using RT-PCR, copy number analysis of selected subclones indicated that clones 2, 3, 6, 8, 9, 11 and 12 had total excision of the loxP cassette (Fig. 3c, left). Additional sequencing analysis confirmed that clone 12 contains a knockout of A_2A_R on both alleles, with one base (T) insertion on one allele and an 18 base deletion on the other (Fig. 3c, right). Clone 12 was selected for line continuation (A2AR KO).

### Development of Stable K562 Cell Lines Overexpressing A_2A_ and A_2B_ Receptors

**Figure 4:**
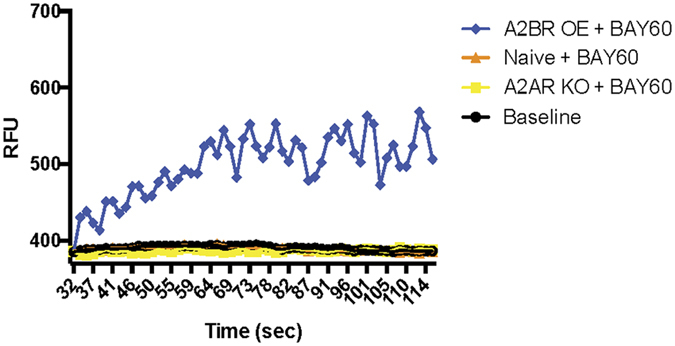
Calcium flux assay for the BAY60 A_2B_R agonist compound shows significant specificity to the A_2B_R receptor. A2AR KO, naïve and A2BR OE cells were treated with BAY60 and release of calcium was measured as relative fluorescence units (RFU). Cells with vehicle addition were used for baseline reads.

**Figure 5:**
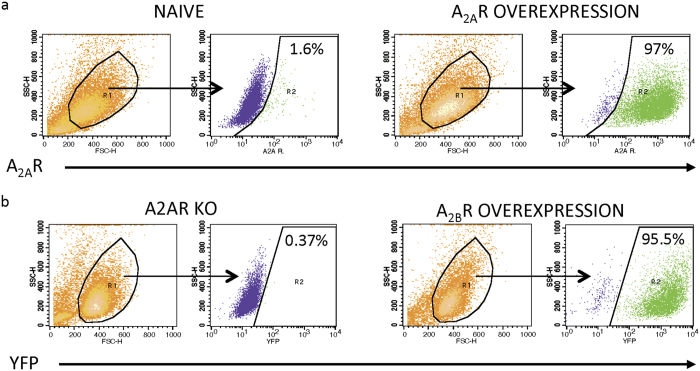
Flow cytometry analysis of engineered A_2A_ and A_2B_ receptor overexpressing cell lines. (**a**) Representative flow cytometry for naïve and A_2A_R overexpressing (A2AR90) K562 cells. (**b**) Representative flow cytometry for A_2A_R knockout (A2AR KO) and A_2B_R overexpressing (A2BR OE) K562 cells (stained for expression of YFP).

Because native K562 cells have very little endogenous expression of the A_2B_R as determined by calcium flux assay (Fig. 4), we did not first knock out the A_2B_R to create the K562 line overexpressing the A_2A_ receptor. The pCL20i4r-EF1a-hgcOPT vector (constructed as described in the Methods) was transduced into K562 naïve cells. Flow cytometry analysis of the stably transduced A_2A_R overexpressing K562 line (A2AR90) shows high expression of A_2A_R in over 97% of transfected cells (Fig. 5a).

To create the A_2B_R overexpressing K562 line (A2BR OE), we transfected our A_2A_R knockout line (created as described above) with the pEYFP-N1-A2BR plasmid, which also expresses YFP upon successful transfection of the A_2B_R. After transfection and neomycin selection (100 ug/ml), the stable A_2B_R overexpressing line showed 95.5% of YFP (Fig. 5b). Since we did not have an A_2B_R antibody, we used the calcium flux assay (described below) to show response to a known A_2B_R agonist as evidence that A_2B_R is being expressed.

### Engineered adenosine cell lines coupled with calcium flux assay can quickly determine specificity of A_2A_ and A_2B_ receptor agonists

To test the efficiency of these cell lines to detect specificity of A_2B_R agonists, we used the well studied A_2B_ receptor agonist BAY60–6583 (BAY60), which is highly selective for the A_2B_R over A_1_, A_2A_ and A_3_ receptors^26^. A_2A_R knockout, naïve and A_2B_R overexpressing cells were plated and subjected to calcium flux assay during BAY60 addition. Using a microplate reader, changes in intracellular free calcium levels upon compound addition were recorded as relative fluorescence units (RFU) over time. No response was seen from naïve or A2AR KO cell lines, while there was significant increase in RFU from A_2B_R overexpressing cells treated with BAY60 (Fig. 4). This experiment confirms that the A2BR OE line overexpresses functional A_2B_R, allowing demonstration of specificity for A_2B_R agonists.

**Figure 6:**
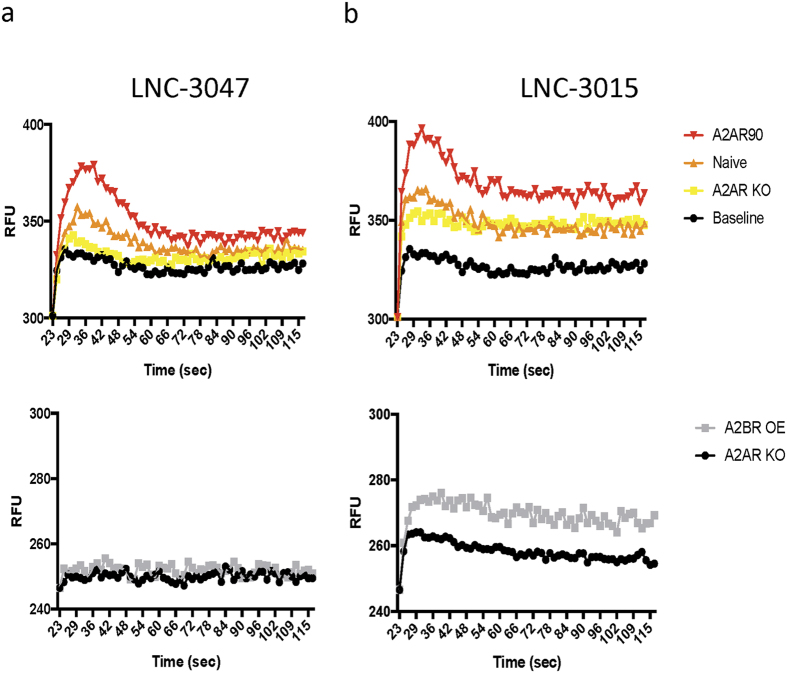
Calcium flux assays for LNC-3047 and LNC-3015, two newly developed A_2A_R agonist compounds, show specificity for LNC-3047 but not for LNC-3015. (**a**,**b**) Top, A2AR KO, naïve and A2AR90 cells were treated with LNC-3047 or LNC-3015 and release of calcium was measured as RFU. Cells with vehicle addition were used for baseline reads. Bottom, comparison of A2AR KO and A2BR OE lines treated with LNC-3047 or LNC-3015.

Our laboratory obtained several newly developed A_2A_R agonists from Lewis and Clark Pharmaceuticals Inc. for testing using our cell lines^27^. Compound LNC-3047 was tested for A_2A_R specificity using calcium release detection. LNC-3047 shows specificity, as calcium release increases proportionally to A_2A_R expression (Fig. 6a). The A2AR90 cell line showed significantly higher release of calcium over the naïve K562 cells treated with LNC-3047. In contrast, the knockout line showed no increase in calcium release over baseline upon addition of compound, indicating that LNC-3047 is highly active in stimulating A_2A_R (Fig. 6a, Top). When the A_2B_R OE line was treated with LNC-3047, there was no calcium release in cells expressing high levels of A_2B_R, indicating that LNC-3047 is very specific to the A_2A_ receptor and does not bind to the A_2B_ receptor (Fig. 6a, Bottom).

A second compound tested, LNC-3015, was shown to be non-specific for the A_2A_R and confirms the specificity, efficiency and utility of our method for distinguishing agonists that are highly specific for A_2A_R versus those with residual A_2B_R activity. LNC-3015, while showing calcium release in both the A2AR90 line and the naïve line, also shows release in the A_2A_R knockout line, indicating non-specific binding to additional receptors (Fig. 6b, Top). When this compound was further screened using the A_2B_R overexpressing line, it can be seen that LNC-3015 causes calcium release in this line as well, indicating that LNC-3015 is also able to bind to the A_2B_R in addition to the A_2A_R (Fig. 6b, Bottom). Overall, these results show that our method using these engineered K562 lines is efficient and fast in identifying non-specific binding of agonists to adenosine receptors.

### Verification of specificity of A_2A_R agonist compounds in cell culture

**Figure 7:**
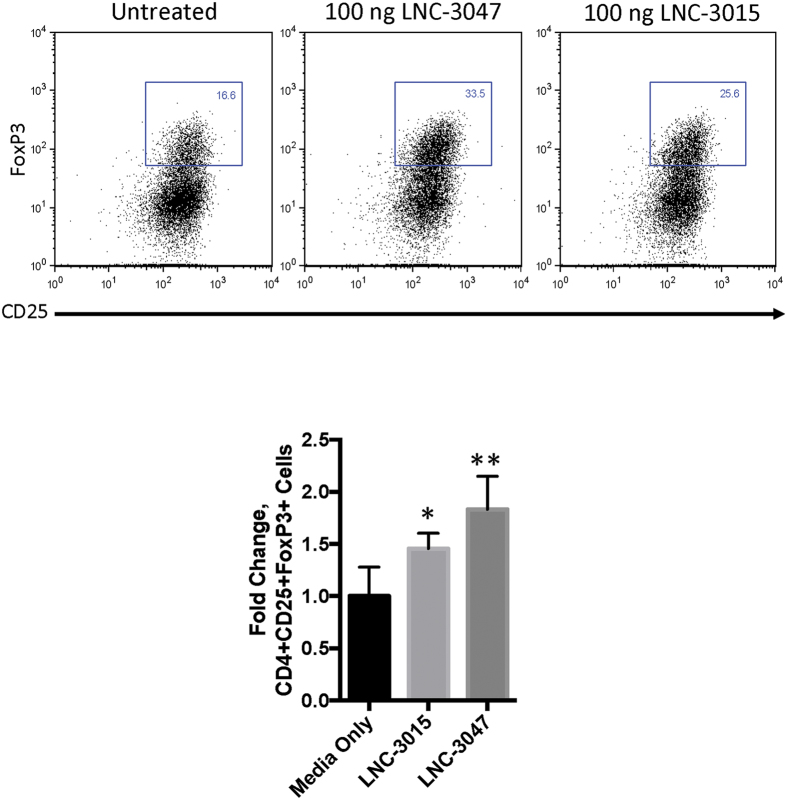
Treatment of naïve CD4 T-cells with LNC agonist compounds induces FoxP3 expression. Representative flow cytometry for treated human CD4 T-cells. Cells were initially gated on CD4^+^CD25^+^, followed by gating for FoxP3^+^ cells. Fold change in FoxP3 expression in cells treated with LNC compounds is shown. Error bars are defined as mean + SEM, n = 5 for all points. Student’s T-test with Mann-Whitney was used to determine significance. *p = 0.0318, **p = 0.0079.

After determining specificity of A_2A_R agonist compounds with calcium flux, these compounds were tested for efficacy in human T-cells. The adenosine 2 A receptor has been shown to play a part in the induction of regulatory T-cells from naïve T-cells, a cell type shown to be important for immune tolerance. Using naïve human CD4^+^CD25^−^ T-cells, LNC-3047 and LNC-3015 were tested for their ability to convert naïve T-cells to FoxP3^+^ regulatory T-cells. Using flow cytometry and gating on viable CD4^+^CD25^+^ cells, untreated cells were found to have 16.6% CD4^+^CD25^+^FoxP3^+^ cells (Fig. 7). Overall, LNC-3047 showed a 2-fold increase in FoxP3 expression (p = 0.0079), while LNC-3015 was around a 1.5 fold increase (p = 0.0317). The reduced efficacy of LNC-3015 may be due to activation of inflammatory pathways by binding of LNC-3015 to the A_2B_ receptor, reducing the differentiation of regulatory T-cells in preference of cytotoxic CD8^+^ T-cells.

Binding data in the form of Ki values obtained from Lewis and Clark Pharmaceuticals Inc. for both LNC-3015 and LNC-3047 confirmed our results (Supplementary Table S1). Ki values are commonly used to predict clinically relevant drug interactions, with smaller Ki values indicating a greater binding affinity of the compound for the receptor^28^,^29^,^30^. Both LNC-3015 and LNC-3047 have much smaller Ki values for the human A_2A_R than CGS-21680 and Regadenoson (Lexiscan), two commonly used A_2A_R agonists that have been shown to be highly specific to the A_2A_R. The selectivity of LNC-3047 for the A_2A_R over both the A_1_ and A_3_ receptors is much higher than that of LNC-3015, supporting our calcium flux and cell culture findings. Together, both the cell culture expression of FoxP3 and Ki binding values validate our calcium flux method for identifying A_2A_ receptor specific agonists.

## Discussion

The use of adenosine agonists has become more prevalent in recent years and is a growing field for many areas of treatment, including cancer, cardiovascular issues and autoimmune diseases^14^,^31^,^32^. Production of agonists specific to individual receptors remains a slow and challenging process and requires the use of expensive and hazardous radioactive reagents. With the development of our new method, we hope to speed up and streamline this process to decrease the time from bench to bedside.

To accomplish this goal, we first developed three distinct new K562 cell lines. Because A_2A_R and A_2B_R share many structural and sequence similarities, we focused on these receptors. We created A_2A_R knockout cells as well as an A_2A_R and A_2B_R overexpressing line. Using these lines, and in conjunction with calcium release, we were able to determine the specificity of several compounds. This method of determining binding specificity of agonists is much quicker, safer and more efficient than the current radio-ligand, binding and competition assays currently used.

The first compound tested, BAY60, is a known A_2B_R agonist. We found that our cell lines were sufficient to determine specificity of this compound, with a significant increase in calcium release from A_2B_R overexpressing cells, but no release over baseline for naïve and A_2A_R knockout cells. Two additional compounds, LNC-3047 and LNC-3015, were expected to be specific to the A_2A_R in human cells. While we did find that LNC-3047 is very specific, LNC-3015 was found to partially bind to the A_2B_R, as seen by RFU increase in A_2B_R overexpressing cells treated with LNC-3015. When these two compounds were tested for efficacy in promoting FoxP3 expression, it was found that LNC-3047 was more efficient at inducing regulatory T-cell differentiation. FoxP3 expression in human cells, while significant, was lessened in cells treated with LNC-3015, most likely due to activation of inflammatory pathways through its binding to A_2B_R.

Our data have shown that our novel method can quickly and efficiently determine receptor-binding specificity for both the A_2A_R and the A_2B_R. In addition, this technology can easily be expanded in the future to test both the A_1_ and A_3_ receptors. Using CRISPR technology, several knockout lines can be generated in K562, jurkat or any other cell type to test a range of compounds. Overall, this method allows for faster and more sensitive testing of new adenosine agonist compounds.

## Methods

### Vectors and Plasmids

HIV-lentiviral vectors were produced by transient transfection of DNA from four separate plasmids into 293 T cells as follows: 20 ug of vector plasmid DNA, 12 ug of pCAG-GP, 4 ug of pCAG-RTR, and 4 ug of pVSV-G plasmid DNA were transfected using Lipofectamine 2000 (Life Technologies, Carlsbad, CA, USA). Cells were transduced with 1X virus and 5 ug/ml of protamine sulfate, followed by spinning at 2,500 rpm for 30 minutes.

To develop the K562 derived A_2A_R overexpressing (A2AR90) line, the lentivector transfer plasmid pCL20i4r-EF1a-hA2aR was created (see Supplementary Fig. S1). This was accomplished by digestion of a previously described lentivector EF1a-hgcOPT for removal of hgc OPT DNA using the EcoRI and NotI enzymes (Thermo Fisher Scientific, Rockville, MD, USA), followed by insertion of the 1.27 kb hA_2A_R cDNA (derived as described below)^33^.

To create the A_2A_R knockout line (A2AR KO), a viral vector was created using the plentiCRISPR loxP plasmid (Supplementary Fig. S1), which was modified from the original plasmid plentiCRISPR V2 (Addgene plasmid # 173707) by addition of a loxP site in the 3′LTR^34^. To accomplish this, the plentiCRISPR V2 plasmid was first cut with PmeI and MluI (Thermo Fisher Scientific) to remove the WPRE (woodchuck hepatitis virus post-transcriptional regulatory element) and SIN (Self-inactivating) 3′LTR. The WPRE and loxP site from pHAGE2Ef1afull-STEM CCA (a plasmid DNA having 4 transcription factors for iPS cells) was amplified with the following primer set (forward primer; 5′-AGTCGATAACGCGTAATCAACCTCTGGATTAC-3′ and reverse primer; 5′-TCCTTAATGTTTAAACTGCTAGAGATTTTCC-3′) using Phusion Polymerase (New England Biolabs, Ipswich, MA, USA) and cloned into the open plentiCRISPR V2 plasmid DNA. Using the BsmBI digestion enzyme (Thermo Fisher Scientific), the plentiCRISPR loxP plasmid was linearized and eluted for insertion of the A_2A_R guide RNA (gRNA). The loxP cassette was removed from stable knockout cell lines using the pcDNA3.1 ZEO T2A CRE-GFP vector (Supplementary Fig. S1).

Finally, for development of the stable A_2B_R overexpressing line (A2BR OE), we used the pEYFP-N1- A_2B_R plasmid, which expresses both the A_2B_R and the eYFP chromophore to simplify selection of transfected clones, a gift from Robert Tarran (Addgene plasmid #37202, Supplementary Fig. S1)^35^.

### RNA Extraction, Reverse-Transcription and qRT-PCR

Human peripheral blood was collected from healthy volunteers after written informed consent under IRB approved NIH clinical protocol 05-I-0213. All methods were carried out in accordance with NIH guidelines and regulations. Human white blood cells were used for RNA extraction using the RNeasy Mini Kit (Qiagen, Hilden, Germany). The A_2A_R cDNA was synthesized following the manufacturer’s protocol by the High Capacity cDNA Reverse Transcription Kit (ABIResearch, New York, NY, USA) using primers (forward primer; 5′-GCCTGCCTGAATTCTGTGGCCATGCCCATCATGGGCTC-3′ and reverse primer; 5′-TTCCCTTAGGCGGCCGCAAACTCCATGAATCATCAGG-3′) for cloning into the pCL20i4rEF1-α backbone. The copy number of the HIV-lentivector gene in DNA isolated from transfected K562 cells was determined by quantitative TaqMan PCR (Applied Biosystems, Foster City, CA, USA), using a multiplex method that allows us to amplify both the HIV-lentivector gene and the human housekeeping gene phenol sulfotransferase (STP) in the same well. The sequence of primers and probe for the HIV-lentivector were; forward primer: 5′-TGAAAGCGAAAGGGAAACCA-3′, reverse primer: 5′-CCGTGCGCGCTTCAG-3′, probe: 5′-FAM-AGCTCTCTCGACGCAGGACTCGGC-TAMRA-3′. The sequence of primers and probe for STP gene were; forward primer: 5′ GGTGCCCTTCCTTGAGTTCA-3′, reverse primer: 5′-CCCCTTGCACCCAGGAC-3′, probe: 5′-VIC-CCCCAGGGATTCCCTCAGGTGTGT-TAMRA-3′. TaqMan PCR conditions were: 50 °C/2 min: 95 °C/10 min: 40cycles, 95 °C/15 sec, and 60 °C/60 sec. Single copy or two copies of HIV-lentiviral vector in K562 cells confirmed by southern blot assay was used for standard curve of transgene copy number.

### Flow Cytometry

Cells were washed and resuspended in serum free and azide free PBS (Life Technologies) and were stained with A_2A_R-FITC, CD4-FITC or CD25-PE for 30 minutes at room temperature. Cells transduced with lentiCRISPR loxP A_2A_R virus were marked using anti-FLAG-FITC antibody (Sigma-Aldrich, St. Louis, MO, USA) after fixing and permeabilization. An isotype control was used for each fluorophore used. For FoxP3 staining, cells were fixed and permeabilized for 30 minutes at room temperature using the eBioscience FoxP3 staining buffer set (Affymetrix, San Diego, CA, USA), followed by staining with anti-human FoxP3-APC (Affymetrix). Samples were run on a benchtop BD FACSCanto flow cytometer (BD Biosciences, San Diego, CA, USA) and a FACScan (BD Biosciences). Analysis was performed using FlowJo FACS analysis software (FlowJo LLC, Ashland, OR, USA) and CellQuestPro (BD Biosciences).

### T7 E1 Assay

Transduced or transfected cell DNA was isolated using the DNeasy blood and tissue kit (Qiagen). From purified DNA, gRNA targeting the A_2A_R region was amplified using Dream Taq Green PCR Master Mix (2X) (Thermo Fisher Scientific). The sequence of primers was; forward primer: 5′-AATGCAGGGAGCCATGGATAGTGCTGG-3′, reverse primer: 5′-AAGATGGAGCTCTGCGTGAGGACCAGG-3′. The PCR product was purified using the GeneJET PCR Purification kit (Thermo Fisher Scientific). The purified PCR product was denatured at 95 °C for 5 minutes and then ramped down to 25 °C at 5 °C/min, followed by cutting with 10 units of T7 E1 enzyme (New England Biolabs) at 37 °C for 1 hour.

### Calcium Flux

Calcium flux assays were performed using the FLIPR Calcium 3 Assay Explorer Kit (Molecular Devices, Sunnyvale, CA, USA). 100 ul of 2.5 × 10^5^ K562 cells were seeded per well in a 96-well plate. An equal volume of Calcium Assay 3 Dye was added to suspended cells and allowed to incubate for 1 hour at 37 °C. Agonist compound solutions were prepared in a separate 96 well plate at a 10X higher concentration than desired final concentration. Plates were then loaded into the FLEXstation 3 Multi-Mode Microplate Reader (Molecular Devices) and allowed to equilibrate for 5 minutes. In conjunction with the SoftMax Pro Microplate Data Acquisition and Analysis Software (Molecular Devices), the FLEXstation added compounds and read the change in fluorescence intensity resulting from calcium release in each well.

### Regulatory T-Cell Activation

Peripheral blood mononuclear cells (PBMCs) were first prepared from human blood by isolation using lymphocyte separation medium (MP Biomedicals, Solon, OH, USA). Human peripheral blood was collected from healthy volunteers after written informed consent under IRB approved NIH clinical protocol 05-I-0213. Possible contaminating red blood cells were lysed using ACK lysis buffer (Quality Biological, Gaithersburg, MD, USA). CD4^+^CD25^−^ T-cells were isolated from whole PBMC using the human CD4^+^CD25^+^ regulatory T-cell isolation kit (Miltenyi, Gaithersburg, MD, USA). Cells were cultured in RPMI (Life Technologies) with 10% fetal bovine serum (Atlanta Biologicals, Flowery Branch, GA, USA) and 100 ug/ml Penicillin/Streptomycin (Quality Biological), activated with CD3/CD28 human T-Activator Dynabeads (Life Technologies) and analyzed 72 hours after plating.

### Statistics

Statistics were performed for all cell culture experiments. Error bars are defined as mean ± SEM. Student’s T-test with Mann-Whitney was used to determine significance as all sample sizes were N = 5 and Mann-Whitney provides a more conservative p-value for small sample sizes.

## Additional Information

**How to cite this article**: Jones, K. R. *et al*. A Novel Method for Screening Adenosine Receptor Specific Agonists for Use in Adenosine Drug Development. *Sci. Rep.* **7**, 44816; doi: 10.1038/srep44816 (2017).

**Publisher's note:** Springer Nature remains neutral with regard to jurisdictional claims in published maps and institutional affiliations.

## Supplementary Material

Supplemental Figure S1 and Table S1

## Figures and Tables

**Figure 1:**
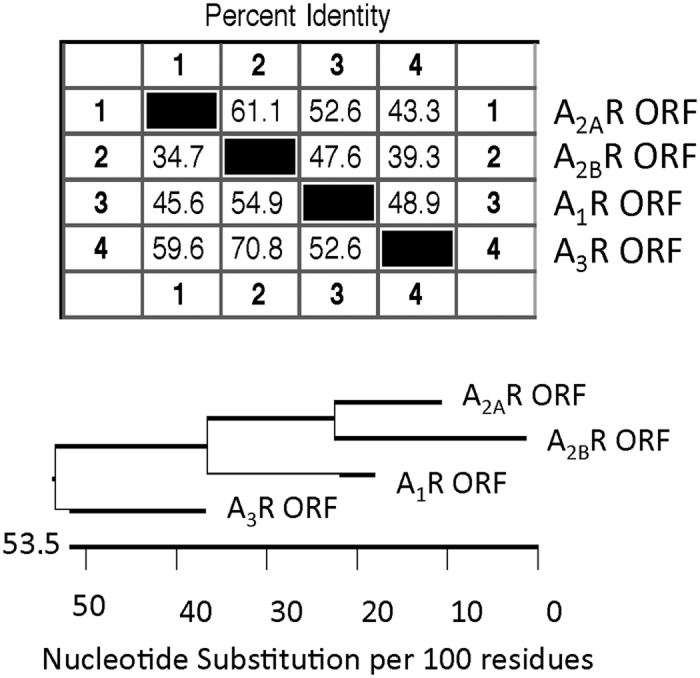
Sequence homology between the four adenosine receptors. The A_2A_ and A_2B_ receptors show the most sequence homology among the adenosine receptors, with these two receptors having a 61% identity.
