# Supplementary material for: A Novel Method for Screening Adenosine Receptor Specific Agonists for Use in Adenosine Drug Development
Source: Sci Rep. 2017 Mar 20;7:44816. doi: 10.1038/srep44816 (PMC5357845; doi:10.1038/srep44816)
Supplement: Supplemental Figure S1 and Table S1 [file srep44816-s1.pdf]

A Novel Method for Screening Adenosine Receptor Specific Agonists for Use in Adenosine Drug Development

Karlie R Jones[1*♯], Uimook Choi[1*], Ji-Liang Gao[2], Robert D Thompson[3], Larry Rodman[3], Harry L Malech[1] and Elizabeth M Kang[1]

*These authors contributed equally to this work

[1]Laboratory of Host Defenses, National Institute of Allergy and Infectious Diseases, National Institutes of Health, Bethesda MD 20892 USA

[2]Molecular Signaling Section, Laboratory of Molecular Immunology, National Institute of Allergy and Infectious Diseases, National Institutes of Health, Bethesda MD 20892 USA

[3]Lewis and Clark Pharmaceuticals, Charlottesville, VA 22901 USA

[♯]Karlie.sharma@nih.gov

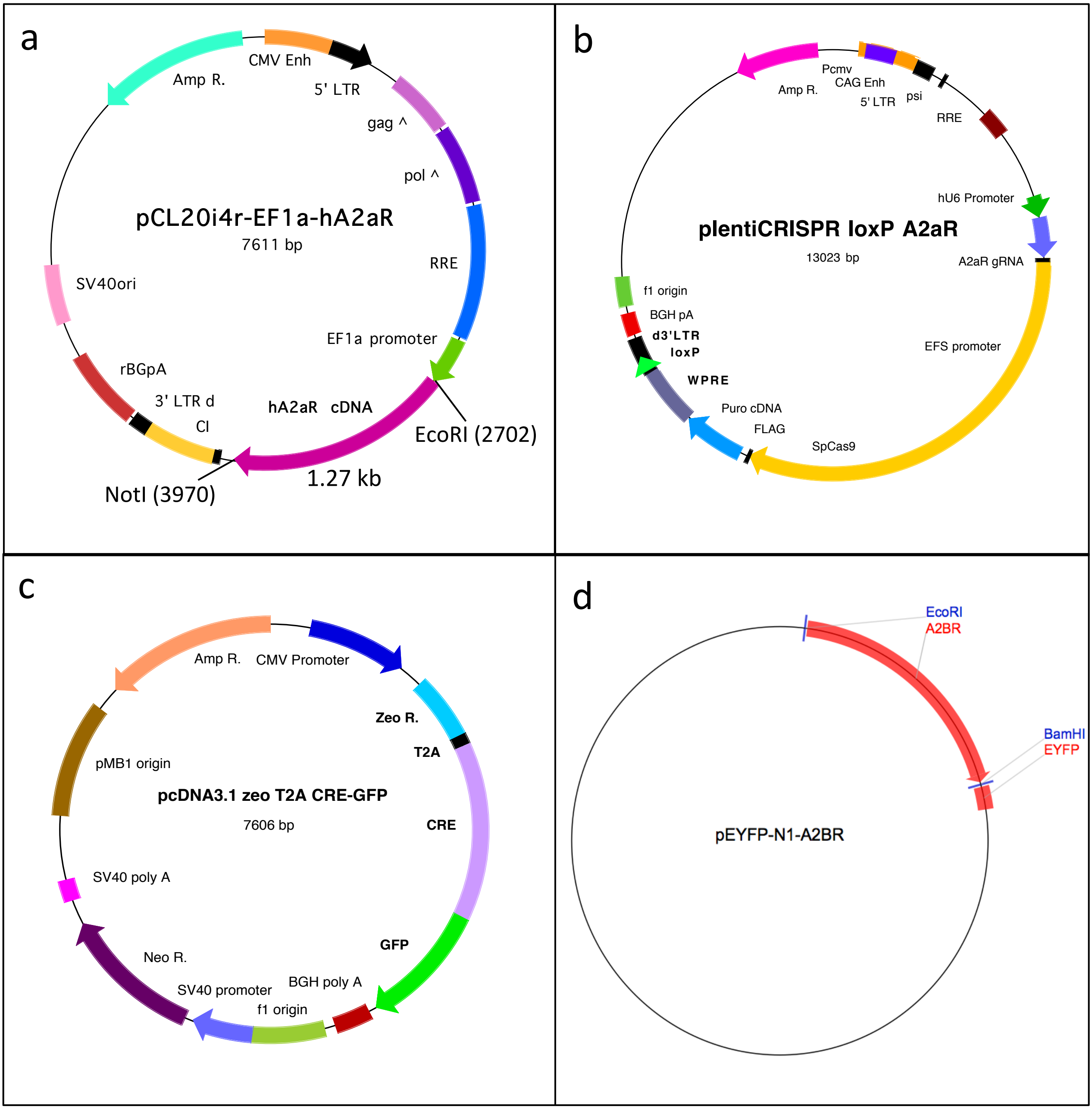

Supplemental Figure S1: Maps for plasmids and vectors used to create knockout and overexpressing cell lines. (a) Map for vector pCL20i4r-EF1a-hA2aR including the 1.27 kb bA2aR cDNA insert. (b) plentiCRISPR loxP A2aR plasmid map. (c) pcDNA3.1 ZEO T2A CRE-GFP vector for the extraction of the loxP cassette from stable knockout clones. (d) Map for the pEYFP-N1-A2BR plasmid, Addgene plasmid #37202.

Compound	A_1R Ki (nM)	$A_{2A}R$ Ki (nM)	A_3R Ki (nM)	Selectivity ($A_1R/A_{2A}R$)	Selectivity ($A_3R/A_{2A}R$)
CGS-21680	316	4.9	82	64	16.73
Regadenoson (CV-3146)	22.7	10.4	417	2.2	40.10
LNC-3015	58.30	0.095	0.39	613.7	4.105
LNC-3047	92.06	0.092	1.52	1000.7	16.566

Supplemental Table S1: Ki values for LNC-3015, LNC-3047, CGS-21680 and Regadenoson (in nM) for human adenosine receptors. CGS-21680 and Regadenoson (Lexiscan) are both commonly used $A_{2A}R$ agonists and have been shown to be specific to the $A_{2A}R$ with Ki values of 4.9 and 10.4, respectively. LNC-3015 and LNC-3047 both have much lower Ki values of 0.095 and 0.092 respectively. Selectivity indicates specificity of a compound for the $A_{2A}R$ over the A_1R or A_3R. LNC-3047 $A_{2A}R$ agonist Ki values are estimates from the functional assay used to determine binding data for LNC-3015.
